# Methylphenidate for attention-deficit/hyperactivity disorder in adults: a narrative review

**DOI:** 10.1007/s00213-021-05946-0

**Published:** 2021-08-26

**Authors:** Rafał R. Jaeschke, Ewelina Sujkowska, Magdalena Sowa-Kućma

**Affiliations:** 1 Section of Affective Disorders, Department of Psychiatry, Jagiellonian University Medical College, ul. Kopernika 21a, 31-501 Kraków, Poland; 2 Department of Human Physiology, Institute of Medical Sciences, Medical College of Rzeszów University, ul. Kopisto 2a, 35-315 Rzeszów, Poland; 3 Centre for Innovative Research in Medical and Natural Sciences, Medical College of Rzeszów University, ul. Warzywna 1a, 35-310 Rzeszów, Poland

**Keywords:** Adult ADHD, Treatment, Psychostimulants, Methylphenidate, Pharmacology

## Abstract

**Rationale:**

Psychostimulants, including methylphenidate (MPH), are the mainstay of pharmacotherapy for attention-deficit/hyperactivity disorder (ADHD) in adults. Even though MPH is the most commonly used medication for ADHD these days, there are relatively few resources available that provide comprehensive insight into the pharmacological and clinical features of the compound.

**Objective:**

The aim of this paper is to provide an up-to-date outline of the pharmacology and clinical utility of MPH for ADHD in adult patients.

**Methods:**

While conducting the narrative review, we applied structured search strategies covering the two major online databases (MEDLINE and Cochrane Central Register of Controlled Trials). In addition, we performed handsearching of reference lists of relevant papers.

**Results:**

Methylphenidate exhibits multimodal mechanism of action, working primarily as a dopamine and noradrenaline reuptake inhibitor. It also protects the dopaminergic system against the ongoing ‘wearing off’ (by securing a substantial reserve pool of the neurotransmitter, stored in the presynaptic vesicles). In placebo-controlled trials, MPH was shown to be moderately effective both against the core ADHD symptoms (standardized mean difference [SMD], 0.49; 95% confidence interval [CI], 0.35–0.64), and the accompanying emotion regulation deficits (SMD, 0.34; 95% CI, 0.23–0.45). The most common adverse events related to long-term treatment with MPH are decreased appetite (~ 20%), dry mouth (15%), heart palpitations (13%), gastrointestinal infections (~ 10%), and agitation/feeling restless (~ 10%).

**Conclusions:**

There is substantial body of evidence to suggest that MPH is an effective and safe treatment option for adults with ADHD.

## Introduction

Attention-deficit/hyperactivity disorder (ADHD) is a common clinical condition, affecting around 1.5–5% of the population globally (Fayyad et al. 2017; Kooij et al. 2019a; Polanczyk et al. 2015). While often misperceived as ‘a mental health problem of the youth’, there is robust evidence to suggest that ADHD is actually a lifelong disorder, of neurodevelopmental origin (Breda et al. 2020; Demontis et al. 2019; Faraone et al. 2021), with the symptoms waxing and waning in contextual fashion (Asherson et al. 2016; Kooij et al. 2019a; Posner et al. 2020). Notably, the estimated point prevalence of ADHD among children and adolescents is quite similar to the corresponding data in adults (3–5% and 1.4–3.6%, respectively) (Fayyad et al. 2017; Polanczyk et al. 2015). Attention-deficit/hyperactivity disorder remains underdiagnosed and undertreated, in one part because of highly prevalent co-existence of other mental health problems (e.g., anxiety disorders, depression, bipolar disorder or personality issues [Katzman et al. 2017]); in other part — due to the overlapping symptoms (notably, emotional dysregulation — making it hard to distinguish between ADHD and borderline personality disorder Beheshti et al. 2020; Moukhtarian et al. 2018).

While the formal diagnostic criteria for ADHD focus on the two major clusters of symptoms (inattention and hyperactivity/impulsivity) (American Psychiatric Association 2013; Reed et al. 2019; Volkow and Swanson 2013), this purely descriptive approach seems to be missing the point (Posner et al. 2020; Wong et al. 2019). From the clinical perspective, treatment for ADHD is primarily about patient-important outcomes, rather than any pre-specified diagnostic measures. Given the trait-like nature of the condition (which is to say that ‘the symptoms [of ADHD] do not reflect a change from the premorbid state’ [Asherson et al. 2016; cited verbatim]), it is vital for a clinician to comprehend the specific areas of functional impairment — as experienced by the individual patient.

Notably, while the above-mentioned areas of disability are certainly related to the ‘narrow’ ADHD symptomatology (see Table 1), they are not mutually equivalent (Boland et al. 2020; Canadian ADHD Resource Alliance [CADDRA] (2018); Joseph et al. 2019; Kooij et al. 2019b). Even though the severity of the core symptoms of hyperactivity/impulsivity (and, to a lesser extent, inattention [Franke et al. 2018]) seems to decrease over time, no similar trend is observed in terms of corresponding impairment in daily functioning (Faraone et al. 2015, 2006). The tangential relationship between the overt ADHD symptomatology on the one hand, and the actual burden of the condition on the other is particularly puzzling, as it sets a specific (and apparently counterintuitive) frame of reference for clinical decision-making. Having said that, one might ask, what does it ‘really’ mean to have ADHD?

**Table 1:** Symptomatology of attention-deficit/hyperactivity disorder and the corresponding areas of functional impairment

*The symptoms*	
Inattention	• Failing to pay close attention to details and making careless mistakes at work or other activities • Difficulty sustaining attention • Seemingly ‘not listening’ when spoken to directly • Often failing to follow through on given instructions and finding it hard to finish chores or duties in the workplace • Having problems organizing tasks and activities • Being reluctant to engage in tasks that require sustained mental effort • Often losing things that are necessary for given tasks or activities • Being easily distracted by irrelevant stimuli or unrelated thoughts (‘wandering mind’) • Often being forgetful in daily activities
Hyperactivity and impulsivity	• Often fidgeting with or tapping hands or feet / squirming in one’s seat • Often leaving one’s seat in situations in which one is expected to remain seated • Often feeling restless (or agitated inside; finding it hard to relax) • Finding it hard to engage in leisure activities quietly • Often ‘being on the go’; acting as if ‘driven by a motor’ • Talking excessively • Often blurting out answers before a question has been completed • Often finding it difficult (frustrating) while awaiting one’s turn (e.g., waiting in line) • Often interrupting or intruding on others
*The likely areas of impairment (with examples)*	
Health problems and psychiatric comorbidities	• Specific learning disorders and executive dysfunctions • Developmental coordination disorders • Speech and language disorders • Mood and anxiety disorders • Autism spectrum disorders • Obsessive–compulsive disorder • Tic disorder • Substance use disorders • Overweight, obesity and related metabolic disorders • Accidents (including driving safety issues) and related injuries • Suicidality
Other reasons for functional impairment	• Undermined sense of well-being (i.e., lower quality of life) • Emotional dysregulation:* − Problems with temper control (i.e., pronounced irritability with frequent, yet short-lived, outbursts of anger) − Emotional over-reactivity (i.e., noticeable problems with managing ‘everyday stressors’, leading to the pervasive sense of being overwhelmed) − Mood lability (i.e., typically swinging from normal mood, to sadness, to mild excitement – and back again…)
Academic and occupational challenges	• Under-performing at school or at work • ‘Staying back’ (e.g., repeating classes), as a consequence of problems related to inattention • Special education needs • School expulsion / dropping out
Social/interpersonal problems	• Inadequate social skills • Long-term pattern of impaired family / intimate relationships

^*^ Reduced ability to modulate the current emotional state in a contextually adequate and goal-directed manner

Adapted from American Psychiatric Association (2013), Volkow and Swanson (2013), Shaw et al. (2014), Faraone et al. (2015), Moukhtarian et al. (2018), Kooij et al. (2019b) and Boedhoe et al. (2020)

In order to address this question in a relevant manner, it is worth beginning with the emphasis on what ADHD is not. Accordingly, the clinical condition discussed in this paper is not actually about (as self-evident as seemingly disjoined) a set of difficulties related to sustaining attention and ‘having hard time sitting still’, or — at the very least — that is just the tip of the iceberg. In fact, there is plenty of data to suggest that the core clinical phenomenon related to ADHD (and the major source of the condition-specific impairment) is the excessive spontaneous mind wandering (MW) (Bozhilova et al. 2018; Christoff et al. 2016). One should also bear in mind that another significant driver behind the ADHD-related impairments are emotion regulation deficits (observed in about 34–70% of adults with ADHD) (Hirsch et al. 2018, 2019).

From the point of view of the MW hypothesis, what tends to be seen ‘from the outside’ as mere attention deficits should be perceived as a by-product of unrestrained variability of thought movement, which — in turn — reflects the diminished ability to ‘suppress internally oriented cognition’ (Christoff et al. 2016; cited verbatim). In plain words, individuals with ADHD are distracted primarily by their own train of thoughts, ever less relevant to the task at hand. Mind wandering typical for ADHD can be, thus, seen as a reflection of insufficient cognitive control mechanisms, overstretched by environmental demands (relentlessly changing in their quality and quantity [Vatansever et al. 2019]). However, this is not the end of the story, as there are some specific features of the ADHD-related MW, making it different from ‘ordinary daydreaming’: the ineffectiveness of context regulation (i.e., the frequency of wandering thoughts is not constrained easily, regardless of the situation), perceptual decoupling (i.e., reduced capacity to respond to external sensory stimuli when ‘roaming mentally’), and a clear sense of relief while engaging in salient and rewarding activities (Bozhilova et al. 2018). Mind wandering has been found to be closely related to all the three dimensions of ADHD symptomatology (i.e., attention deficits, hyperactivity, and impulsivity) — as well as the corresponding emotional dysregulation. At the same time, the intensity of MW seems to be independent from the history of traumatic brain injuries, substance use disorders, educational deficits, or perinatal adverse outcomes — further substantiating the idea that the phenomenon of spontaneous MW is the specific, (dys)functional hub of ADHD (Biederman et al. 2019).

The MW hypothesis is particularly important from the clinical perspective, because it makes is easier to see ADHD-related challenges for what they really are. Actually, the experience of ADHD can be compared to the predicament of a chess player immersing oneself in endless analyses, while finding it very hard to make any ‘real-life’ decisions regarding the moves on the board — and ultimately losing on time. Notably, the MW perspective also provides some important insights into the pathogenesis of the condition. Not only is mind wandering the key predictor of the level of impairment related to ADHD (Biederman et al. 2019; Bozhilova et al. 2018; Helfer et al. 2019), it also seems to be a direct consequence of some specific neural disruptions. As described in the seminal paper by Bozhilova et al. (2018), ‘the MW hypothesis proposes that altered interaction between the four large scale networks (default mode network [DMN], executive control network, salience network and visual network), and that deficient DMN deactivation during task activities will lead to excessive spontaneous MW, lacking in coherence and topic stability, which in turn will lead to ADHD symptomatology’ (cited verbatim). Overall, ADHD is nowadays considered to be primarily a ‘brain connectivity disorder’ (Demontis et al. 2019; Michelini et al. 2019; Sudre et al. 2018); the evidence on the structural correlates of the condition is less certain (for details, see a series of reports from neuroimaging studies by the ENIGMA-ADHD Working Group: Boedhoe et al. [2020]; Hoogman et al. [2017], [ 2019] and Zhang-James et al. [2021]).

Psychostimulants (methylphenidate [MPH], dexamphetamine, and lisdexamphetamine) are the mainstay of pharmacotherapy for ADHD in adults (Bolea-Alamañac et al. 2014a, b). Even though MPH is the most commonly used medication for ADHD these days (Raman et al. 2018), there are relatively few resources available that provide comprehensive insight into the pharmacological and clinical features of the compound. Therefore, the aim of this paper is to deliver a concise overview of the current knowledge on the utility of MPH in the population of adults diagnosed with ADHD.

## Methods

While it is not a formal systematic review, we applied structured search strategies covering the two major online databases (PubMed / MEDLINE and Cochrane Central Register of Controlled Trials; for the MeSH terms, see Table 2). In order to confine the findings to the reliable clinical data (thus applying the idea of ‘new evidence pyramid’ [Murad et al. 2016]), we focused primarily on high-quality systematic reviews (Murad et al. 2014) and treatment guidelines (Brouwers et al. 2010; Shekelle 2018). Following the recent methodological advice by Faltinsen et al. (Faltinsen et al. 2019), we considered observational studies as potentially valuable addition to randomized controlled trials (RCTs) on ADHD in adults.

**Table 2:** Search strategy

PubMed/MEDLINE	Cochrane Central Register of Controlled Trials
*For clinical data:* (("Methylphenidate"[Mesh]) AND "Attention Deficit Disorder with Hyperactivity"[Mesh]) AND "Adult"[Mesh] *For pharmacological data:* ( "Methylphenidate/metabolism"[Mesh] OR "Methylphenidate/organization and administration"[Mesh] OR "Methylphenidate/pharmacokinetics"[Mesh] OR "Methylphenidate/pharmacology"[Mesh] OR "Methylphenidate/physiology"[Mesh] OR "Methylphenidate/poisoning"[Mesh] OR "Methylphenidate/toxicity"[Mesh])	"methylphenidate hydrochloride" in Title Abstract Keyword AND "ADHD" in Title Abstract Keyword AND adult* in Title Abstract Keyword—with Cochrane Library publication date Between Jan 2018 and Sep 2020 (Word variations were searched)

Bearing in mind that the notion of hierarchy of medical evidence applies primarily to clinical practice, we applied more inclusive criteria while searching for pharmacological studies (Table 2). In addition, we performed handsearching of reference lists from articles identified in the online databases.

## Methylphenidate: chemistry and general pharmacology

First synthesized in 1944, the compound nowadays known as MPH belongs to the class of phenylethylamines and is chemically identified as methyl-2-phenyl-2-(piperidin-2-yl)acetate. The molecular formula of MPH is C_14_H_19_NO_2_ and its mass equals 233.31 g/mol (Lange et al. 2010; Wenthur 2016). There are four configurational isomers of MPH, with the *d*-threo enantiomer being the most active pharmacologically (Dinis-Oliveira 2017; Markowitz et al. 2003a). The earliest formulations of MPH (as approved by the U.S. Food and Drug Administration [FDA]) were rapidly released and absorbed into the bloodstream, and then metabolized just as quickly. Significant progress in the techniques of drug formulation (especially over the previous two decades) resulted in the launch of new pharmaceutical preparations including various enantiomers of MPH, with distinct pharmacokinetic profiles (including extended-release formulations), or dosage forms (Childress et al. 2019; Markowitz et al. 2003a). It was a watershed moment in the history of treatments for ADHD, since the availability of a wide range of MPH formulations enables for ‘tailoring’ of the treatment regimens to the individual needs of the patients (notably, this aspect seems to be particularly relevant for adults [Kooij 2013]).

Methylphenidate was identified as a psychostimulant already in 1954, but to this day, the mechanism by which it exerts behavioral effect has not been fully elucidated. However, it is assumed that antagonism against the dopamine (DA) and noradrenaline (NA) transporters (DAT and NAT, respectively) plays the pivotal role (Cortese 2020; Lange et al. 2010).

### An overview of the specific formulations of methylphenidate

**Fig. 1:**
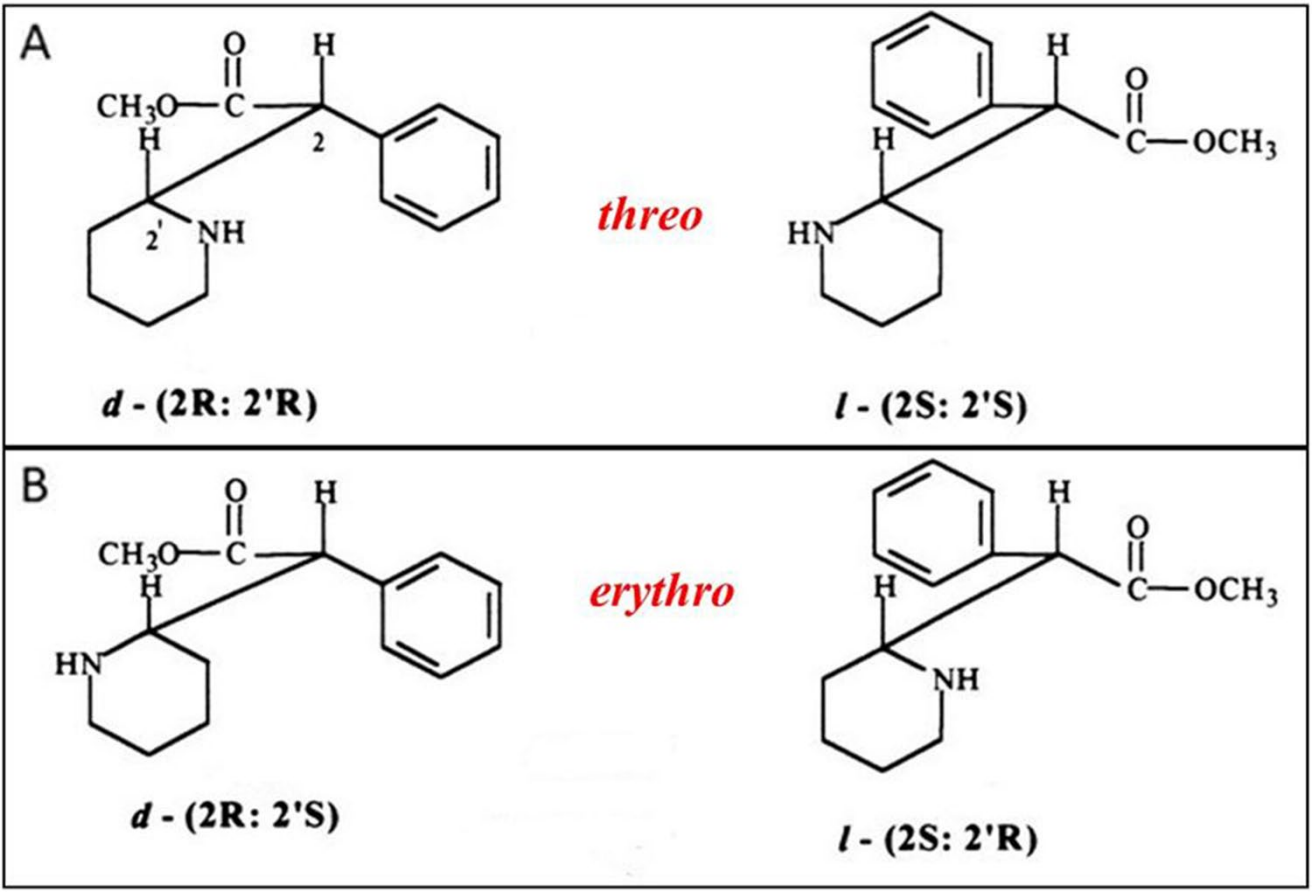
Structures of the four configurational (and two stereo-) isomers of the methylphenidate (Adapted from Markowitz et al. (2003a))

Due to the fact that an MPH molecule contains two chiral carbon atoms in its structure, it can exist as four isomers, which are usually divided into two pairs of enantiomers: erythro [*d-* (2R:2’R) and *l-* (2S:2’S)) and threo [*d-* (2R:2’R) and *l-* (2S:2’S)) (see Fig. 1). The early formulations introduced in the market in the 1950s included a mixture of both racemates (80% *(d/l)-*erythro and 20% *(d/l)-*threo) (Bartl et al. 2017; Heal and Pierce 2006; Markowitz et al. 2003a). As it became clear that the central stimulating effects of methylphenidate are associated with the activity of threo isomers administration, while erythro isomers cause some adverse side effects, the production of more purified preparations became the subject of interest (Childress et al. 2019; Markowitz et al. 2003a). Therefore, subsequent generations of the drug contained only the threo enantiomers in equal proportions between the *d-*(2R:2’R) and *l-*(2S:2’S) forms of the molecule (which is the case for the majority of the currently available formulations). Finally, high-purity formulations containing *d*-threo-MPH only (the most potent form of the drug) were developed. The drug containing only this isomer is sometimes called *d*-TMP, although other names, dexmethylphenidate, D-MPH, or D-threomethylphenidate, are used more commonly in the literature (Dinis-Oliveira 2017; Markowitz et al. 2003a) Fig.2.

The history of clinical use of MPH started with the immediate-release (IR) formulations. However, due to their rapid metabolism, and thus the need to apply them multiple times a day, many patients find the treatment regimen not very comfortable (Childress et al. 2019; Kooij 2013; Maldonado 2013; Markowitz et al. 2003a; Patrick et al. 2019; Swanson et al. 1999, 2003). As a response to this challenge, the sustained-release (SR) and — later on — extended-release (ER) formulations were developed (Markowitz et al. 2003a). Nowadays, the mainstay of treatment for ADHD are the ‘long-acting/dual release’ MPH capsules, combining IR and ER formulations in varying proportions (IR 20–50% and ER 50–80%; see Table 3). This ingenious resolution ensures both the rapid onset of action and durability of the clinical effects (for details, see Childress et al. 2019 and Kooij 2013).

**Table 3:** An overview of the available formulations of methylphenidate

Brand name	Formulation and dosage form	Doses available
Adhansia XR®	Methylphenidate hydrochloride; extended-release capsules	25, 35, 45, 55, 70, 85 mg
Aptensio XR™	Methylphenidate hydrochloride; extended-release capsules	10, 15, 20, 30, 40, 50, 60 mg
Concerta®	Methylphenidate hydrochloride; extended-release tablets	18, 27, 36, 54 mg
Cotempla XR-ODT™	Methylphenidate extended-release orally disintegrating tablets	8.6, 17.3, 25.9 mg
Daytrana®	Methylphenidate transdermal patch	10 mg / 9 h (1.1 mg/h) 15 mg / 9 h (1.6 mg/h) 20 mg / 9 h (2.2 mg/h) 30 mg / 9 h (3.3 mg/h)
Focalin™	Dexmethylphenidate hydrochloride; immediate-release tablets	2.5, 5, 10 mg
Focalin XR™	Dexmethylphenidate hydrochloride; extended-release capsules	5, 10, 15, 20, 25, 30, 35, 40 mg
Jornay PM®	Methylphenidate hydrochloride; extended-release capsules	20, 40, 60, 80, 100 mg
Metadate CD®	Methylphenidate hydrochloride; extended-release capsules	10, 20, 30, 40, 50, 60 mg
Methylin®	Methylphenidate hydrochloride; immediate-release oral solution	5 mg/5 ml, 10 mg/5 ml
Methylin ER®	Methylphenidate hydrochloride; extended-release chewable tablets	10, 20 mg
Quillichew ER®	Methylphenidate hydrochloride; extended-release chewable tablets	20, 30, 40 mg
Quillichew XR®	Methylphenidate hydrochloride; extended-release oral suspension	5 mg/ml
Ritalin®	Methylphenidate hydrochloride) immediate-release tablets	5, 10, 20 mg
Ritalin LA®	Methylphenidate hydrochloride) extended-release capsules	10, 20, 30, 40 mg

*Additional remarks:*

1. This overview covers the FDA-approved (and currently available) medications, containing methylphenidate as the active ingredient, including available strengths, formulation and dosage form. Most of them are for oral use (except Daytrana® – transdermal patches) and contain a mixture of *d/l* enantiomers (except Focalin XR™ – *d*-MPH)

2. Numerous generics under the name methylphenidate hydrochloride and dexmethylphenidate hydrochloride were omitted from the list

*ER/XR* extended release, *FDA* U.S. Food and Drug Administration, *LA* long-acting, *MPH* methylphenidate

Adapted from Childress et al. (2019) and FDA (Food and Drug Administration 2004, 2010a, b, 2017a, b, c, d, 2019)

In addition to the ‘classical’ oral tablets and capsules, chewable tablets are also available. The extensive product range is complemented by oral solutions or suspensions and transdermal patch (Cortese et al. 2017; Wenthur 2016) (for details, see Table 3). The wide variety of available formulations, using various drug delivery systems (e.g., osmotic-release oral system MPH: OROS MPH; methylphenidate transdermal system: MTS, and MPH extended-release oral suspension: MEROS), as well as diverse composition (in terms of the proportions of both the *d/l* enantiomers and the content of the IR:ER formulations within the tablet/capsule), contribute to the better acceptability of the treatment (as compared to the ‘simple’ IR formulations of MPH) (Childress et al. 2019; Wenthur 2016).

### Pharmacokinetic profile of methylphenidate

Regardless of the type of formulation, the active component of the medication is the highly soluble salt: MPH hydrochloride (Childress et al. 2019).

Following oral administration, MPH absorbs quickly and almost completely from the gastrointestinal tract and buccal mucosa (Patrick et al. 2005). Low pH inhibits the non-enzymatic hydrolysis of MPH; hence, the gastric juice probably only slightly decomposes it. However, due to a large first-pass effect, the absolute bioavailability (F) is low and in humans oscillates between 0.11 and 0.53, while in monkeys and rats, the corresponding values equal are to 0.22 and 0.19, respectively (Chan et al. 1983; Kimko et al. 1999; Markowitz et al. 2003a; Wargin et al. 1983; Wenthur 2016). The peak plasma concentration (C_max_) depends primarily on the type of formulation and shows high interindividual variability (see Table 4).

**Table 4:** Pharmacokinetic properties of some of the FDA-approved formulations of MPH in healthy adults (except for Daytrana ®; see the footnote)

Brand name	Dose tested	Dosage	Food	PK profile	PK parameters				References
					AUC_0-inf_ (ng/ml*h^−1^)	T_max_ (h)	C_max_ (ng/ml)	T_1/2_ (h)	
					Mean ± SD or median (min. – max.)				
Adhansia XR ®	100 mg	Multiple (once daily for 5 days)	Fasting	2 peaks	AUC_0-24:_ 227.17 ± 83.61	T_max1:_ 1.5(1–2.5) T_max2:_ ~ 12 (8.5–16.0)	15.73 ± 4.54	~ 7	Food and Drug Administration 2019
Concerta ®	18 mg	Single		1 peak	41.8 ± 13.9	6.8 ± 1.8	3.7 ± 1.0	3.5 ± 0.4	Food and Drug Administration 2017a
Cotempla XR-ODT ™	51.8 mg	Single		1 peak	169.1 ± 57.13	4.98 (2.5 – 6.5)	20.8 ± 5.22	4.0 ± 0.73	Food and Drug Administration 2017b
Daytrana ® ^1^	12.5 cm^2^/9 h (10 mg/9 h)	Single		1 peak	48.7 ± 21.9	10.0 (6.00 – 12.0)	4.15 ± 2.59	4.35 ± 0.788	Childress et al. 2019; Food and Drug Administration 2017c
Focalin XR™^2^	20 mg	Single		2 peaks	119.1 ± 40.7	T_max1:_ 1.5 (1 – 2.0) T_max2:_ 6.5 (4.5 – 7.0)	C_max1:_ 13.7 ± 4.6 C_max2:_ 14.9 ± 4.0	3.26 ± 0.51	Food and Drug Administration 2004
Ritalin LA ®	20 mg	Single		2 peaks	45.8 ± 10.0	T_max1:_ 2.0 ± 0.9 T_max2:_ 5.5 ± 0.8	C_max1:_ 5.3 ± 0.9 C_max2:_ 6.2 ± 1.6	3.3 ± 0.4	Food and Drug Administration 2010b
Methylin ®	2 mg/ml	Single	Fasting	1 peak	51.91 ± 24.73	1.707 ± 0.444	9.391 ± 3.002	2.955 ± 0.602	Food and Drug Administration 2010a
			Satiety		64.95 ± 25.21	2.667 ± 0.747	10.693 ± 2.639	2.897 ± 0.663	
Methylin ER ®	20 mg	Single	Fasting	1 peak	41.19 ± 7.71	4.17 ± 0.95	4.59 ± 0.79	4.38 ± 1.30	Food and Drug Administration 2017d
			Satiety		51.12 ± 9.69	4.38 ± 1.09	6.64 ± 1.19	3.21 ± 1.00	

^1^ Daytrana ® — results drawn from a study on ADHD in adolescents

^2^ Focalin XR ™ is the only product containing solely *d*-MPH; the other formulations are the mixtures of *d/l*-MPH

*PK profile/parameters* pharmacokinetic profile/parameters, *ADHD* attention-deficit/hyperactivity disorder, *AUC*_*0-inf*_ area under the concentration – time curve to infinite time, *C*_*max*_ peak plasma concentration after drug administration, *ER/XR* extended-release, *FDA* U.S. Food and Drug Administration, *MPH* methylphenidate, *SD* standard deviation, *T*_*max*_ time to reach C_max_, *T*_*1/2*_ half-life

In the case of the IR formulations, there is consistent research data to suggest that time to reach C_max_ (T_max_) ranges from 1 to 3 h, and the onset of action is already observable about 20 min after the ingestion of the drug (Spiller et al. 2013). On the other hand, the corresponding evidence pertaining to the ER formulations of MPH is much more heterogeneous (which reflects significant differences in terms of the specific release systems, as designed and used by individual manufacturers) (Cortese et al. 2017). For example, after oral administration of OROS MPH, the plasma concentrations of the drug increase rapidly, reaching an initial maximum at ~ 1 h, followed by gradual ascending concentrations over the next 5 to 9 h. Then, a gradual decrease in concentration is observed. Regardless of the dose, T_max_ for OROS MPH oscillates between 6 and 10 h (McNeil Pediatrics 2007), while T_1/2_ is about 2.6–3 h (Leonard et al. 2004). When taken during a meal, MPH reaches the C_max_ about 1 h later (Spiller et al. 2013) (see Table 4). It was also reported that the fat content in the meal may either accelerate or slow down the absorption of MPH, with no major impact on other pharmacokinetic parameters (Chan et al. 1983; Markowitz et al. 2003a; Modi et al. 2000b). The effects of fat-rich meals on the C_max_ value may differ substantially (of note, this remark applies primarily to some ER formulations). Both combined IR/ER and OROS MPH formulations are characterized by lower absorption ratio, while the liquid-based ER MPH exhibits elevated levels of C_max_, when ingested with fatty meals (Food and Drug Administration 2017b; 2018).

Methylphenidate is highly soluble in lipids and shows low (15%) plasma protein binding; therefore, it is rapidly distributed to various tissues (in rats: kidney > lungs > brain > heart > liver) (Dinis-Oliveira 2017; Kimko et al. 1999; Patrick et al. 2005; Volkow et al. 1995; Wolraich and Doffing 2004). The concentration of the drug measured in the brain was shown to be approximately eight times higher than the corresponding value detected in the blood, regardless of the route of administration (intravenous vs. oral) (Patrick et al. 2005). A positron emission tomography (PET) study in humans showed that 7.5% (± 1.5%) of [^11^C]-MPH molecules (administered intravenously) enter the brain, with the highest concentration detected in the striatum, and significantly lower in the cortex and cerebellum (Food and Drug Administration 2017b; Volkow et al. 1995). In a subsequent study performed by the same team of researchers, it was found that various MPH isomers (*l*- and *d*-threo) are distributed differently within the brain (Ding et al. 1997). Accordingly, the maximum regional uptake of [^11^C]-*d*-threo-MPH was detected in the basal ganglia, while the uptake ratio of [^11^C]-*l*-threo-MPH was similar across the brain. The volume of distribution ratio for the basal ganglia to cerebellum (DVB/DVC) at the steady-state was ranging between 2.2 and 3.3 for [^11^C]-*d-*threo-MPH in baboons and humans, and was equal to 1.1 for [^11^C]-*l*-threo-MPH (Ding et al. 1997). In baboons, pretreatment with unlabeled MPH led to significant decrease of [^11^C]-*d-*threo-MPH uptake in the striatum, but not in the cerebellum. At the same time, no changes in DVB/DVC ratio after [^11^C]-*l-*threo-MPH administration were observed (Ding et al. 1995). Also, a microdialysis study in rats indicated markedly increased extracellular dopamine levels (by about 650%) after *d*-threo-MPH administration (which was not the case for *l*-threo-MPH) (Ding et al. 1997).

The above-mentioned findings suggest that pharmacological specificity of MPH resides entirely on the *d*-threo isomer. They also indicate that binding of the *l*-isomer in the human brain is mostly nonspecific.

Methylphenidate undergoes fast systemic clearance (as calculated by Markowitz et al. 2003b, the oral clearance ratio is approximately 4.5 L/kg/h), with little or no accumulation of the drug ‘from day to day’ — regardless of the formulation used (Modi et al. 2000a, 2000c; Rochdi et al. 2004). That is why, treatment with MPH keeps on following an ‘on–off mode’ (with typical late afternoon rebounds of ADHD symptoms [Kooij 2013]), rather than reaching a stable ‘plateau’ (pharmacokinetic or clinical) (Patrick et al. 2005). At the same time, it needs to be emphasized that the fast systemic clearance of MPH is coupled (somewhat paradoxically) with the slow clearance from the brain (Volkow et al. 1995).

In general terms, there are no major differences in the pharmacokinetic parameters of MPH between children, adolescents, and adults (Cortese et al. 2017; Markowitz et al. 2003a; Wenthur 2016). On the other hand, there is some variation in these parameters between men and women. For example, Patrick et al. (2007) observed a lower bioavailability of MPH in blood of women (*n* = 10) with a simultaneously stronger stimulating effect compared to the group of men (*n* = 10). Interestingly, brain studies found consistently higher concentrations of MPH in female rats than in males, which contributed to a consistently higher exposure of the brain to the drug. The observed gender differences may be a consequence of the different metabolic rate of the drug. There was a significant decrease in the clearance of MPH in the brain of females as compared to males, especially in the case of the *d-*enantiomer (Bentley et al. 2015). Other factors, such as the rate of drug transport across the blood–brain barrier, genetic and hormonal factors, or the influence of the immune system, may also play a role. Unfortunately, the role of these putative mechanisms has so far been poorly understood and requires further research (Kok et al. 2020).

Methylphenidate is metabolized in the liver, mainly by endoplasmic reticulum human carboxylesterase 1A1 (CES1A1) through a de-esterification process into the inactive metabolite phenyl-2-piperidine acetic acid, also known as (either *d*- or *l*-threo) ritalinic acid (Childress et al. 2019). The process described here is enantio-selective, which implies significantly higher plasma concentration and longer T_½_ of *d*-MPH in comparison to *l*-MPH (because CES1A1 has six-fold higher preference for *l*-MPH versus *d*-MPH) (Dinis-Oliveira 2017). Notably, CES1A1 is closely related to CES2; however, MPH is metabolized by CES1 only (Stevens et al. 2019). In studies on various oral formulations of MPH, it was shown that AUC_0-inf_ for the *l* enantiomer is equal to just 1–15% of the AUC_0-inf_ value for *d*-MPH (Patrick et al. 2005). Despite the fact that *l*-MPH has a lower bioavailability, it is likely that this enantiomer is more stable in human plasma than *d*-MPH, while less stable in human erythrocytes (Ramos et al. 1999; Srinivas et al. 1991). The rule of enantio-selectiveness also applies to the trans-esterification of MPH, which occurs after co-administration of MPH with ethanol (Markowitz et al. 2000, 1999). There are some preliminary reports suggesting that the product of the trans-esterification (known as ethylphenidate – EPH) may actually be toxic (Dinis-Oliveira 2017).

In addition, hydroxylated metabolites of MPH have also been detected in a number of preclinical studies (involving various animal species). Of note, some of the compounds (e.g., para-hydroxy-MPH) exhibit pharmacological activity in mice (effect not studied in humans), even greater than that of MPH (Wenthur 2016). Para-hydroxy-MPH undergoes further de-esterification and glucuronidation and gives the final inactive product: *p*-hydroxy-ritalinic acid glucuronide.

As a consequence of microsomal oxidation, 6-oxo-methylphenidate (another inactive metabolite of MPH) may be formed, which is further converted to 6-oxo-ritalinic acid via de-esterification (Stevens et al. 2019).

Methylphenidate is eliminated as ritalinic acid (approximately 60–80% of a dose taken), predominantly with urine. After oral administration, 50% of a dose of methylphenidate is excreted in the urine within 8 h and 90% by 48 h post-administration (Wolraich and Doffing 2004). About 1–3% of the dose administered orally is excreted in feces, and less than 1–2% is eliminated unchanged in urine (Childress et al. 2019; Stevens et al. 2019).

### Drug-drug interactions

Only a few clinically significant pharmacokinetic interactions involving MPH have been identified so far (Childress et al. 2019; Schoretsanitis et al. 2019).

Since the kinetics of MPH release is pH-dependent (this remark refers mostly to the XR formulations), a co-administration with gastric acid modulators may alter its release, pK profile, and pharmacodynamics. Hence, a simultaneous use of proton pump inhibitors (e.g., omeprazole, esomeprazole, pantoprazole) or H_2_-blockers (e.g., famotidine) or antacids (e.g., sodium bicarbonate) is not recommended (Food and Drug Administration 2017b, 2019). Also, the mechanisms of active drug release from individual formulations can also be severely altered by concomitant alcohol consumption. It was reported that most of the long-acting formulations show a much faster release of the active ingredient when co-administered with beverages containing ≥ 40% of alcohol (Childress et al. 2019). The interaction of ethanol with *d/l*- or *d*-MPH increased the C_max_ by 22% and 15%, respectively, and was linked to stronger stimulating effects of MPH (Zhu et al. 2017).

Due to the fact that *d/l*-MPH does not significantly inhibit any of the cytochrome P450 enzymes (1A2, 2C8, 2C9, 2C19, 2D6, 2E1 and 3A isoforms), the metabolism of other xenobiotics and the detoxification processes are not particularly disturbed during its intake. For example, the co-administration of MPH and desipramine (a CYP2D6 substrate) did not increase the plasma level of this antidepressant. As mentioned above, MPH is hydrolyzed mostly by the CES1A1 enzyme. Therefore, MPH should be cautiously used in patients who are simultaneously treated with some strong CES1A1 inhibitors. Specifically speaking, the four drugs (aripiprazole, perphenazine, thioridazine, fluoxetine) have relatively high affinity to the above-mentioned enzyme (with the half maximal inhibitory concentration [IC_50_] values of 5.7 µM, 13.9 µM, 7.0 µM, 6.1 µM, respectively), which results in a significant increase in the plasma concentration of MPH (if co-prescribed) (Dinis-Oliveira 2017; Zhu et al. 2010).

To some extent, the metabolism of MPH may be also inhibited by alcohol. Ethanol is known to diminish the activity of CES1A1, while enhancing the transesterification process. As a result, EPH is formed, the presence of which is associated with an increased concentration of *d*-MPH in the blood plasma and increased euphoric effect in humans (Childress et al. 2019).

According to Schoretsanitis et al. (the authors of a recent systematic review [Schoretsanitis et al. 2019]), polytherapy with MPH and strong inducers of drug metabolizing enzymes (with carbamazepine as the prime example; but the interactions between MPH and phenobarbital, phenytoin or rifampin cannot be excluded either) may lead to significant decrease in the blood concentration of MPH. On the other hand, co-administration of imipramine might lead to increase of the bioavailability of MPH. Therefore, the authors concluded that MPH should not be prescribed simultaneously with the above-mentioned medications.

### Pharmacodynamics

The pharmacological activity of MPH results primarily from its direct inhibition of the dopamine and noradrenaline transporters (DAT and NAT, respectively), which is due to its partial similarity of the basic structure to catecholamines. In vitro studies suggest that MPH is characterized by particularly high affinity (independent of the model used) for DAT (solute carrier family 6A member 3 — SLC6A3), slightly lower to NAT (solute carrier family 6A member 2 — SLC6A2), with virtually no effect on the activity of the serotonin transporter (SERT; also known as solute carrier family 6A member 4 — SLC6A4) (Markowitz et al. 2006; Markowitz and Patrick 2008; Williard et al. 2007). It was reported that MPH has almost 1300 times greater affinity for NAT, and about 2200 times greater affinity for DAT, in comparison to SERT (Stevens et al. 2019). Noteworthy, there is considerable variation in the strength of MPH binding to both DAT and NAT, depending on the enantiomer used (Markowitz and Patrick 2008). Accordingly, *d*-MPH was found to exert the highest affinity (DAT: IC_50_ = 23 nM, K_i_ = 161 nM; NAT: 39 nM, K_i_ = 206 nM), *d/l*-MPH mixture exhibited the intermediate binding potency (DAT: IC_50_ = 20 nM, K_i_ = 121 nM; NAT: 51 nM, K_i_ = 788 nM), and the *l*-enantiomer had the least effect on the activity of the transporters (DAT: IC_50_ = 1600 nM, K_i_ = 2250 nM; NAT: IC_50_ > 10^4^ nM, K_i_ > 10^4^ nM) (Riddle et al. 2007; Sandoval et al. 2002; Williard et al. 2007) (for a review, see also Markowitz and Patrick 2008).

Methylphenidate was shown to affect the redistribution of vesicular monoamine transporter-2 (VMAT-2; solute carrier family 18 member 2 — SLC18A2), which is involved in the sequestration of cytoplasmic dopamine and noradrenaline, and thus is an important regulator of neurotransmission. Two studies showed that administration of MPH caused a decrease in VMAT-2 immunoreactivity in the membrane-associated fraction, an increase in the cytoplasmic fraction, and no change in the total synaptosomal pool. These results confirm that MPH does not affect the global amount of VMAT-2 in presynaptic terminals, but only its trafficking (Riddle et al. 2007; Sandoval et al. 2002). The maximum effect of MPH was observed 1 h after administration and returned to control values after 2 h. Notably, the MPH-induced redistribution of VMAT-2 was observed only in monoaminergic neurons (as opposed to either cholinergic, GABA-ergic or glutamatergic cells) (Riddle et al. 2007).

Taken together, MPH increases the availability of DA (by inhibiting the DAT), while protecting the dopaminergic system against the ongoing ‘wearing off’ (by securing a substantial reserve pool of the neurotransmitter, stored in the presynaptic vesicles) (Fleckenstein et al. 2009; German et al. 2015). Those features are highly relevant from the clinical point of view, as they seem to translate into the relatively low risk of neurotoxic (or neuropsychiatric, in broader sense) side effects in patients treated with MPH (Fleckenstein et al. 2009; Krinzinger et al. 2019).

**Fig. 2:**
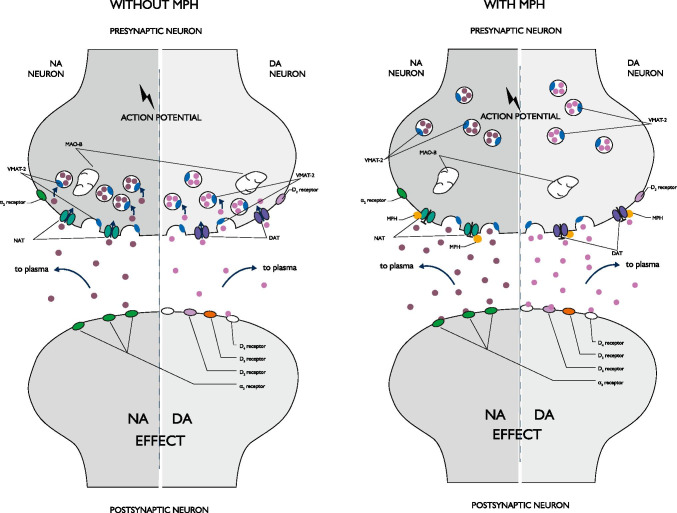
An outline of the mechanisms of action of methylphenidate

The overall outcome of the mechanisms described above is the boost of dopaminergic transmission in the brain, by extending the residence time (and activity) of impulse-released DA in the synaptic cleft (see Fig. 2). Studies on the effects of MPH as a DAT blocker are quite hard to conduct, because of the heterogeneity of dopaminergic receptors. Although MPH has not been shown to have any affinity for dopamine receptors, it is likely to influence their function as well (Stevens et al. 2019). It is suggested that inhibition of dopamine reuptake by MPH reduces dopaminergic activity through increased stimulation of presynaptic inhibitory autoreceptors. Methylphenidate appears to bind to DAT (which blocks the access to the impulse-released catecholamine), but lacks the intrinsic activity necessary to induce the conformational change required for the transporter’s shift (translocation) into the cytoplasm. Therefore, MPH does not influence the cytoplasmic transporter-facilitated release of dopamine into the synaptic cleft when the DAT reverts to its previous conformation (Patrick et al. 2019). Elevated dopamine levels following the MPH intake result in increased availability of the neurotransmitter, and its binding to both dopamine transporters and receptors (Faraone 2018). As suggested by some positron emission tomography (PET) studies, MPH in therapeutic doses blocks more than 50% of DAT and significantly increases the levels of extracellular DA in the basal ganglia, and this effect is modulated by the rate of DA release. In a sample of unmedicated patients with ADHD, an elevated binding of radiolabeled DA analogues in the striatum was reported (a finding suggestive for increased density of DAT molecules, as well). On the other hand, treatment with MPH reduced the number of the available DAT binding sites, and this was correlated with the alleviation of ADHD symptoms. In addition, some variability between DA inhibition and extracellular dopamine levels was observed, suggesting that MPH enhances the basal activity of the dopaminergic system (which remains in line with the hypothesis linking ADHD to diminished responsiveness of dopaminergic neurocircuitry) (Markowitz and Patrick 2008). Actually, administration of MPH leads to a three- to fourfold increase of both DA and NA (in the striatum, as well as the prefrontal cortex) (Hodgkins et al. 2012). By increasing dopaminergic activity in the brain, MPH elevates the overall activity of the central nervous system, with a number of significant behavioral and cognitive effects (for a review, see the article by Kapur 2020).

In a number of magnetic resonance imaging (MRI) studies, it was found that long-term administration of psychostimulants (including MPH) may reduce structural and functional abnormalities observed in the brains of individuals with ADHD (Costa et al. 2013; Frodl and Skokauskas 2012; Hart et al. 2013; Moeller et al. 2014; Mueller et al. 2014; Schlösser et al. 2009; Schweitzer et al. 2004; Spencer et al. 2013; Tomasi et al. 2011) (for a review, see the paper by Faraone 2018).

Although most research on the pharmacological effects of MPH in ADHD is focused on the dopaminergic system, there is a growing body of evidence pointing to the significance of the noradrenergic component as well. Not only has MPH a relatively high affinity for NAT, but it also directly interacts with noradrenergic receptors (Andrews and Lavin 2006; Besnard et al. 2012; Furini et al. 2017; Gamo et al. 2010; Huang et al. 2018). Accordingly, it was reported that MPH binds to several subtypes of α-adrenergic receptors: α_2A_ (K_i_ = 5.6 µM), α_2B_ (K_i_ = 2.420 µM), and α_2C_ (K_i_ = 0.860 µM) (Wenthur 2016). The procognitive effects of MPH (as related to the stimulation of the cerebral cortex) are hypothesized to be mediated by the interactions with the above-mentioned receptors (Andrews and Lavin 2006; Furini et al. 2017; Gamo et al. 2010).

It is not clear whether MPH has affinity for any serotonin receptors. Although the first study showed that both the *d* and *l* enantiomers have some binding potential for both 5-HT_1A_ and 5-HT_2B_; however, the next one only confirmed significant activity of *d*-MPH on 5-HT_1A_, but not 5-HT_1B_ receptors (Markowitz et al. 2006, 2009). Confusingly enough, another study on the potential affinity of MPH (in concentrations < 10 µM) towards either 5-HT or cholinergic receptors returned negative results (Besnard et al. 2012).

## Clinical considerations

### Issues of efficacy and effectiveness

The most comprehensive overview of the efficacy of MPH in adults to date has been provided in the systematic review with network meta-analysis by Cortese et al. (2018). Having synthesized the data collected from participants of 51 RCTs (overall, 8131 patients), the authors of this formidable piece of scientific work came up with a hierarchy of pharmacological treatments for ADHD. Accordingly, in terms of the impact on the core ADHD symptomatology, MPH was found to be significantly more effective than placebo, with the value of effect size remaining within the moderate range (as implied by the standardized mean difference of about 0.5 [Murad et al. 2014]). In comparison to active treatments, MPH turned out to be marginally-to-moderately less efficacious than amphetamines (including lisdexamphetamine), while exhibiting similar efficacy to atomoxetine and bupropion. Finally, modafinil was inferior to MPH. At the same time, the risk of leaving the study because of treatment-related adverse events was comparable across the groups (see Table 5).

**Table 5:** Efficacy and tolerability of MPH over the period of 12 weeks, as compared to placebo or other drugs used for ADHD in adults (adapted from Cortese et al. [2018])

*Comparator agent*	*Efficacy data*		*Tolerability data*	
	SMD (95% CI)	Certainty of evidence*	OR (95% CI)	Certainty of evidence*
Amphetamines	– 0.29 (from – 0.54 to – 0.05)	⊕ ⊕ ◯◯	1.36 (0.54–3.43)	⊕ ◯◯◯
Atomoxetine	0.04 (from – 0.14 to 0.23)	⊕ ⊕ ⊕ ◯	0.97 (0.47–2.02)	⊕ ⊕ ◯◯
Bupropion	0.04 (from – 0.38 to 0.45)	⊕ ⊕ ◯◯	1.07 (0.13–8.92)	⊕ ◯◯◯
Modafinil	0.65 (0.19–1.11)	⊕ ⊕ ◯◯	0.60 (0.19–1.92)	⊕ ◯◯◯
Clonidine	No data available		No data available	
Guanfacine	No data available		No data available	
Placebo	0.49 (0.35–0.64)	⊕ ⊕ ⊕ ◯ moderate	2.39 (1.40–4.08)	⊕ ⊕ ⊕ ⊕

The primary outcomes for efficacy and tolerability were, respectively, the clinician-rated severity of ADHD core symptoms and the proportion of participants who left the study due to any adverse event. Results in bold are statistically significant

The respective SMD cut-off scores of 0.2, 0.5, and 0.8 denote small, moderate, and large clinical effects (Murad et al. 2014)

SMD > 0 favours MPH; OR > 0 favours comparator

^*^ GRADE Working Group grades of evidence (cited verbatim from Balshem et al. [2011]):

High certainty (⊕ ⊕  ⊕ ⊕): We are very confident that the true value of outcome importance lies close to that of the estimate

Moderate certainty (⊕ ⊕  ⊕ ◯): We are moderately confident in the estimate: the true value of outcome importance is likely to be close to the estimate, but there is a possibility that it is substantially different

Low certainty (⊕ ⊕ ◯◯): Our confidence in the estimate is limited: the true value of outcome importance may be substantially different from the estimate

Very low certainty (⊕ ◯◯◯): We have very little confidence in the estimate: the true value of outcome importance is likely to be substantially different from the estimate

*CI* confidence interval, *GRADE* Grading of Recommendations Assessment, Development and Evaluation, *MPH* methylphenidate, *OR* odds ratio, *SMD* standardized mean difference

Considering the broader picture emerging from the above-mentioned systematic review, three issues come to the fore. First: speaking of the treatment choices for ADHD in adults, a ‘one size fits all’ approach does not work — as the drugs differ in terms of efficacy and tolerability profiles. This is a valuable implication, both from the point of view of rank-and-file practitioners and clinical guidelines developers (of note, there has been little consensus among experts about the optimal strategies for sequential pharmacological treatments with psychostimulants and other drugs for ADHD; see Arnett and Stein 2018; Cortese 2020 and Wong et al. 2019). Second: there is a dearth of longer-term trials on pharmacotherapy for ADHD in adults (an important obstacle, since the duration of the therapy in real-life settings usually reflects the chronic course of the condition [Fredriksen et al. 2013]). Third: the certainty of the evidence is relatively low (in other words, the relative effectiveness and safety of the drugs for ADHD has not been determined yet [Balshem et al. 2011]). Having said that, one might ask, is there anything to mention about the clinical effects of the MPH use, beyond the short run?

In fact, there is a growing body of evidence on risks and potential benefits of long-term treatment with MPH in adult patients with ADHD. Accordingly, the authors of the recently published COMPAS trial (The Comparison of Methylphenidate and Psychotherapy in Adult ADHD Study; a multi-centre RCT with over 400 participants) found that after 1 year of treatment, MPH ‘maintained’ the advantage over placebo in a number of efficacy outcomes (Philipsen et al. 2015). Interestingly enough, the COMPAS team found some evidence for the relative stability of this effect over time, as the termination of treatment did not ‘cancel out’ entirely the difference between MPH and placebo at the follow-up of 1.5 years (Lam et al. 2019). Nevertheless, this encouraging conclusion should be seen against the backdrop of the broader line of research on maintenance of response and risk of relapse upon discontinuation of MPH. Data from some other RCTs suggest, accordingly, that therapeutic effects of psychostimulants tend to be rather fragile — with quite rapid re-emergence of ADHD symptoms once the medication is tapered off (Buitelaar et al. 2015; Huss et al. 2014; Tamminga et al. 2021).

In a recent systematic review of pharmacoepidemiological studies, Chang et al. (2019) found that various modalities of drug therapy for ADHD (involving MPH, other stimulants or atomoxetine) may lead to significant improvements in a number of functional outcomes (i.e., risk of injuries, road traffic accidents and substance use disorders; the therapy was also beneficial in terms of educational achievements). Nevertheless, the picture of the long-term effects of the treatment was not that clear. The authors suggested that studies using within-individual design (i.e., making it possible to compare the clinical status of patients while ‘on medication’ vs. the periods of ‘being unmedicated’ or receiving treatment with other classes of drugs — e.g., antidepressants) would facilitate the research on the effectiveness of long-term therapies for ADHD. Unfortunately, this methodological approach has not been widely utilized so far (Chang et al. 2019).

Of particular note, there is emerging evidence to suggest that therapy with psychostimulants (including MPH) is linked to lower rates of antisocial behaviors (Lichtenstein et al. 2012), as well as lower risk of suicide among patients with ADHD (across the age spectrum, from children to middle-aged adults) (Chang et al. 2020). In a meta-analysis of data from 21 placebo-controlled RCTs, MPH was also found to alleviate the ADHD-related emotion regulation deficits, as experienced by adult individuals (SMD, 0.34; 95% CI, 0.23–0.45) (Lenzi et al. 2018).

While the issue of gender differences in terms of MPH effectiveness has been poorly investigated thus far, there is some preliminary data implicating that MPH might be somewhat less efficient in women (Kok et al. 2020; Quinn et al. 2017). Nevertheless, the gender differences (if any) are subtle, and — as such — were not taken into account in the recent treatment guidelines (Young et al. 2020).

### Titration and dosage of methylphenidate

The issue of MPH dose optimization in adults with ADHD is tricky — for a number of reasons.

The confusion begins with the notion of ‘optimal treatment’ in the context of adult ADHD. As pointed out by Huss et al. (2017), there are two (mutually exclusive) meanings of the ‘optimal dosage’ with regard to the medications for the condition discussed: either ‘the dose above which there is no further improvement’, or ‘the lowest dose necessary to achieve optimal therapeutic response’. Bearing in mind the marked individual differences in terms of the dose–response pattern (most likely reflecting the pharmacogenetic diversity of patients with ADHD [Bonvicini et al. 2016; Chermá et al. 2017; National Institute for Health and Care Excellence 2018]), coupled with plethora of formulations of MPH (either currently available [Mattingly et al. 2020] or in the pipeline [Cortese et al. 2017]), it is plausible that the art of the treatment with MPH goes beyond ‘fine tuning of the dosage’. More than anything else, it has to be tailored to the individual needs and expectations of the patient (Buitelaar et al. 2015; Kooij et al. 2019a, 2010).

Accordingly, it is useful to start with the assumption that the action of the drug should be synchronized with daily habits of the person. In other words, the optimal treatment regimen should cover approximately 12–16 h a day (Kooij et al. 2010). Since none of the MPH formulations available these days actually meet this criterion, patients often find it beneficial to combine a long-acting MPH with a shorter-acting ‘version’ of the drug. Bearing in mind significant individual differences with respect to optimal dose requirements, titration of MPH is essentially a process of trial-and-error (Cortese 2020; Huss et al. 2017; Kooij 2013; Volkow and Swanson 2013).

There is general consensus among experts that the strategic goal of treatment with MPH is to make sure that the medication works all day long (in order to make the patient less likely to experience rebound symptoms of ADHD, once MPH wears off) (Cortese 2020; Faraone et al. 2015; Kooij 2013; Mattingly et al. 2020). The tactics, however, should be flexible. In this respect, duration and onset of action of the available MPH formulations (see Table 6) are the key variables to be considered while tailoring the treatment plan (Mattingly et al. 2020). Pragmatically speaking, using long-acting MPH twice daily (around 8 a.m. and 15 p.m.) is the ‘cornerstone’ of pharmacotherapy for adults with ADHD, while shorter-acting formulations might be considered as add-on treatments, making it less likely for a patient to develop rebound symptoms at bedtime. Notably, some patients may find it beneficial to take low doses of short-acting MPH in the evening, in order to become more peaceful and, hence, more likely to fall asleep (Kooij 2013; Mattingly et al. 2020).

**Table 6:** An outline of dosing strategies for methylphenidate formulations (approved by the FDA for ADHD in adults)

Brand name	Dosing (times per day)	Onset of effect	Overall duration of effect	Dose range/provisional maximum dose
Short-acting				
Ritalin®	2–3	1–2 h	4 h	10–60 mg in divided doses Provisional maximum dose: 150 mg/day
Methylin®	2–3	1 h	4 h	
Medikinet CR®	2	5–8 h		
Intermediate-acting				
Methylin ER®	2–3	n.a	n.a	n.a
Ritalin SR®	1	1.5 h	8 h	n.a
Metadate ER®	1	n.a	8 h	n.a
Long-acting				
Quillichew ER®	1	45 min	8 h	Provisional maximum dose: 150 mg/day
Concerta®	1–2	The immediate-release component ⇒ the first peak plasma concentration: 1–2 h following the ingestion The slow-release component ⇒ the second peak plasma concentration: about 7 h following the ingestion	7–12 h	
Quillivant XR®	1	45 min	12 h	
Aptensio XR™	1	1 h	12 h	
Jornay PM®	1	8–10 h	> 12 h	
Adhansia XR®	1	1 h	13–16 h	

*ADHD* attention-deficit/hyperactivity disorder, *ER/XR* extended release, *FDA* U.S. Food and Drug Administration, *n.a.* no data available, *SR* sustained release

Adapted from Banaschewski et al. (2006), Kooij (2013) and Mattingly et al. (2020)

In quantitative terms, no optimal titres of MPH have been determined so far (Huss et al. 2017). According to the recommendations by the British Association for Psychopharmacology, ‘careful titration and monitoring of side effects is required’ (Bolea-Alamañac et al. 2014a, b). More specifically, with regard to the MPH-IR formulation, it was recommended to start with 5 mg twice daily (or equivalent for MPH-ER / OROS), followed by daily or weekly increases (provided that the medication is well tolerated by the patient). In order ‘to make the trial-and-error more informed’ in terms of titration, some ideas can be borrowed from the field of child and adolescent psychiatry — in particular, from the Dundee ADHD Clinical Care Pathway (DACCP). The DACCP framework is based on a highly structured algorithm, utilizing standard protocols for a routine clinical evaluation of patients with ADHD. As reported by Coghill and Seth (2015), meticulous measurement of ADHD symptoms and adverse events significantly improves the quality of clinical decision-making process — thus making the titration of MPH notably smoother. In this regard, a ‘4-week, structured dose-optimization schedule’ is used for all patients prescribed immediate-release stimulants or extended-release methylphenidate. The dose is increased from 5 to 20 mg three times per day for immediate-release formulations or equivalent dose for long-acting formulations. Medication is usually initiated with 12-h cover, 7 days a week, without routine drug holidays’ (Coghill and Seth 2015; cited verbatim).

While no similar strategy has been devised for adults so far, one might expect the titration process to be less straightforward — e.g., because of co-morbidities and (above all) due to significant variability of the individual walks of life, with corresponding differences in terms of the treatment expectations (Steinbuchel and Greenhill 2020). However, speaking of adjusting the doses of MPH in adults, it is noteworthy that every 10-mg dose increase corresponds with the clinical improvement of about 0.11–0.12 (as expressed with the SMD values) (Castells et al. 2011). This means that the generic rule of ‘start low, go slow’ does not necessarily apply to the praxis of therapy with MPH, as the low-to-moderate portions of the medication might turn out to be not effective enough for a significant proportion of adult patients with ADHD (Retz and Retz-Junginger 2014).

Even though there is a considerable body of evidence for the linear dose–response relationship (Cortese et al. 2018), the maximum dose of MPH has not been established as yet. The rule of thumb has it that patients should not be prescribed with MPH at doses exceeding 150 mg/day (Kooij 2013). At the same time, it is worth emphasizing that the ‘red line’ for further titration is marked by any of the three factors (Coghill et al. 2019; Steinbuchel and Greenhill 2020):

1. No room for additional clinical improvement
2. Intolerable adverse events
3. The fact that the maximum dose limit has already been achieved.

### Tolerability and safety: general issues

In terms of tolerability, a Cochrane review of 11 short-term trials (Epstein et al. 2014) suggests that the loss of appetite with subsequent weight decrease is probably the most significant side effect of IR formulations of MPH. Insomnia, headache, dry mouth, tremor, sweating, anxiety, late-afternoon depression, and irritability were also reported. Individuals receiving long-acting MPH are likely to experience a similar range of adverse events, with nasopharyngitis, headache, decreased appetite, dry mouth, and nausea being the most prevalent symptoms (Ginsberg et al. 2014a). There were also significantly higher rates of insomnia in patients receiving OROS MPH (Adler et al. 2011), as compared to modified-release long-acting formulations (20.7 vs. 3.7%) (Ginsberg et al. 2014a).

Methylphenidate seems to be relatively safe in the long run, as well. As observed in the oft-cited placebo-controlled COMPAS study, the following side effects were significantly more prevalent in the MPH sample over the 52 weeks of monitoring: decreased appetite (22 vs. 3.8%), dry mouth (15 vs. 4.8%), heart palpitations (13 vs. 3.3%), gastrointestinal infections (11 vs. 4.8%), agitation (11 vs. 3.3%), feeling restless (10 vs. 2.9%), hyperhidrosis, tachycardia, weight loss (for each of the three outcomes the difference with the placebo group was 6.3 vs. 1.9%), depressed mood (4.9 vs. 1.0%), influenza (4.9 vs. 1.0%), and acute tonsillitis (4.4 vs. 0.5%) (Kis et al. 2020).

While the above-mentioned non-specific adverse events are usually transient and relatively mild (Kooij et al. 2010), their implications for day-to-day clinical practice are subject to debate, fuelled by higher rates of treatment discontinuation due to side effects among patients receiving MPH (in comparison to placebo) (Castells et al. 2013). However, speaking about insomnia, there are reports indicating that there may be a significant relationship between the dose and/or formulation of MPH (or the dosing schedule) and the likelihood of difficulties falling asleep (Stein et al. 2012; Wynchank et al. 2017).

Methylphenidate was repeatedly shown to increase heart rate and blood pressure (Coghill et al. 2013; Epstein et al. 2014; Martinez-Raga et al. 2013), yet it remains unclear whether adult individuals treated with MPH are at significantly higher risk of serious cardiovascular events (Martinez-Raga et al. 2013; Westover and Halm 2012). While Habel et al. (2011) found no evidence for increased risk of myocardial infarction (MI), sudden cardiac death (SCD), or stroke, a corresponding trial by Schelleman et al. (2012) reported significantly higher rates of SCD/ventricular arrhythmias and stroke in a sample of patients treated with MPH (in comparison to individuals who had not used the medication). However, the absolute risk increase (ARI) values were very small (0.2–0.4%; see Table 7). There is also early evidence suggesting that MPH may act as a trigger for arrhythmias, particularly in patients with congenital heart diseases (Shin et al. 2016).

**Table 7:** Risk of serious cardiovascular events in methylphenidate users (adapted from Schelleman et al. [2012])

Outcome	Number of events		RR (95% CI)	ARI (95% CI) *
	Individuals treated with MPH (*N* = 43,999)	Individuals not treated with MPH (*N* = 175,955)		
SCD / VA	54	432	2.73 (2.02–3.70)	0.4%
Stroke	54	796	1.40 (1.05–1.87)	0.2%
MI	47	928	1.09 (0.81–1.48)	NS
Composite outcome of MI or stroke	98	1642	1.25 (1.01–1.54)	0.2%
All-cause death	854	7467	2.31 (2.14–2.56)	6% (5%–6%)

*ARI* absolute risk increase, *CI* confidence interval, *MI* myocardial infarction, *NS* not significant, *RR* relative risk, *SCD* sudden cardiac death, *VA* ventricular arrhythmia

^*^ As calculated by one of the co-authors of this paper (RRJ)

While the legal status of psychostimulants as controlled substances is ‘firmly established’ (e.g., see schedule II of the U.S. Controlled Substances Act [Preuss et al. 2020]), it remains unclear whether in clinical settings MPH should be considered a ‘genuine’ — even if only potential — drug of abuse. Notably, the authors of the two long-term naturalistic studies did not find any significant relationship between treatment with psychostimulants (including MPH) and subsequent substance use disorders in adults with ADHD (Biederman et al. 2008; Faraone et al. 2007) (for an overview, see the article by Fredriksen et al. [2013]). This conclusion remains in line with the seminal paper by Volkow and Swanson (2003), who noticed that abuse liability of MPH depends primarily on the route of administration — with expeditious rise of DA levels, once the medication is snorted or injected (admittedly, these scenarios are very remote from the real-life context of providing treatment for patients with ADHD; see the section ‘Acute toxicity’ below). Quite the opposite, oral formulations of MPH ensure gradual increase of dopaminergic activity, thus keeping ‘slow but sure’ pace of the sustained response of DA-releasing neurons and — coupled with slow clearance of MPH from the brain — making it unlikely to develop a vicious circle of ‘dopaminergic hits followed by the misery of craving’. In other words, the oral formulations ensure slow entry of MPH into the brain — the hallmark of therapeutic utility, instead of abuse liability. Since there is no dose–response relationship between the medication intake and feeling ‘high’ following the intake of oral preparations of MPH (due to the slow increase of the drug concentration in the brain — as opposed to the aftermath of either snorting or injecting MPH for recreational purposes), oral formulations are considered safe in terms of the risk of developing addiction (Volkow and Swanson 2003).

Nevertheless, the authors of the recent pharmacovigilance studies performed in Denmark (Pottegård et al. 2013) and France (Pauly et al. 2018) detected a long-term, upward trend in MPH dose adjustments (with the mean ‘peak’ of 89.6 mg daily among the cluster of 25- to 49-year-old participants of the French cohort [Pauly et al. 2018]). It remains to be determined whether these findings reflect a build-up of tolerance to MPH.

In terms of other potential safety issues, it is worth mentioning that treatment with MPH probably does not yield significant risk of developing psychosis (Chang et al. 2019; Moran et al. 2019). Speaking of a broader range of long-term neuropsychiatric outcomes, the authors of the recent ADDUCE (Attention Deficit / Hyperactivity Disorder Drugs Use Chronic Effects) study found that the data available is as heterogenous as inconclusive. While the treatment with MPH does not seem to elevate the risk of some common psychiatric symptoms (i.e., depressed mood, anxiety and irritability), ‘studies exploring dosing and timing of MPH are warranted in this area’ (Krinzinger et al. 2019; cited verbatim). Also, the available evidence does not support the hypothesis linking therapy with psychostimulants to higher risk of tic disorders among patients with ADHD (Krinzinger et al. 2019) (even though MPH might ‘exacerbate tics in individual cases’; for details, see the Cochrane review by Osland et al. 2018).

Let us close this passage with an important practical note: in cases of ADHD coexisting with hypertension, hyperthyroidism, glaucoma or some cardiac issues (i.e., angina, arrhythmias and hypertrophic cardiomyopathy), neither MPH nor other stimulants should be considered the drugs of choice. In fact, the above-mentioned medical conditions are deemed relative contra-indications for treatment with psychostimulants (Kooij et al. 2010).

### Acute toxicity

There is limited data on acute toxicity of MPH in humans. However, the available evidence suggests that the risk of life-threatening adverse events among individuals who overdosed MPH is very low (Bruggisser et al. 2011; Hondebrink et al. 2015; Rietjens et al. 2017).

The most commonly reported signs of MPH overdose are drowsiness, tachycardia, dry mouth, headache, and agitation (Hondebrink et al. 2015). This finding is consistent with theoretical considerations suggesting that MPH toxicity represents a sympathomimetic syndrome driven by ‘excessive blockade’ of NAT, leading to overstimulation of both α- and β-adrenergic receptors (Spiller et al. 2013). Therefore, from the clinical standpoint, dopaminergic mechanisms behind MPH toxicity seem to be less important in comparison with the noradrenergic components (Rietjens et al. 2017).

In the vast majority (that is, around 90%) of cases, symptoms related to MPH toxicity are mild, transient, and self-limiting. More severe outcomes (e.g., psychosis or arrhythmia) are much rarer (Hondebrink et al. 2015). Nevertheless, once they develop, the patient should be treated just like any other individual who overdosed stimulants (in other words, the general rules for clinical management of amphetamine poisoning do apply) (Spiller et al. 2013). Having said that, the MPH dose threshold for hospital referral remains subject to controversy — making it unclear ‘who actually requires evaluation at the emergency department’. The postulated cut-off scores suggestive for clinically significant MPH intoxication vary between 2 and 3 mg/kg (as discussed in depth by Hondebrink et al. 2015 and Scharman et al. 2007); also, the laboratory alert level for the MPH blood concentration was recently set at 50 ng/ml (Hiemke et al. 2018). As a rule of thumb, all patients who developed hallucinations, abnormal movements, or chest pain subsequently to MPH ingestion should be referred to a hospital for further evaluation (Scharman et al. 2007). The quantitative (laboratory) measures of the presumed MPH toxicity seem to be much less certain, as there is no clear dose–effect relationship in this case (Rietjens et al. 2017) and, first and foremost, the optimal therapeutic range of MPH doses has not been determined yet (Cortese 2020; Cortese et al. 2018) (see ‘Discussion’).

Of particular note, the route of administration of MPH is an important determinant of the risk of poisoning. Accordingly, there is evidence to suggest that MPH, if administered intravenously, may lead to some severe toxic reactions (e.g., local ischemia and skin necrosis). It seems to be the case that individuals who abuse MPH in oral or nasal forms are not subject to the risks of that kind (Bruggisser et al. 2011).

### Tolerability and safety of methylphenidate in specific populations

#### The elderly

There is very limited data regarding safety of MPH among older adults. However, the authors of a recently published observational study (encompassing 113 individuals aged ≥ 55 years, who were receiving treatment for ADHD at a specialized outpatient clinic in the Netherlands) found that the risk-to-benefit did not seem to differ from the population of younger adults. Nevertheless, the results need to be replicated in rigorously designed and meticulously conducted RCTs (Michielsen et al. 2020).

#### Pregnant or breastfeeding women

Even though there is an increasing trend of prescribing stimulants during pregnancy and breastfeeding, safety data for this practice is scarce (Bolea-Alamanac et al. 2014; Haervig et al. 2014; McAllister-Williams et al. 2017). This is why, as a precaution, MPH and related medications are not recommended for pregnant or breastfeeding women (Young et al. 2020).

While MPH does not seem to have teratogenic properties, there are concerns regarding risk of miscarriages, cardiac malformations, deceleration of fetal growth, as well as preterm birth and placenta-associated pregnancy complications (e.g., preeclampsia). However, the available evidence is largely inconclusive, since the absolute risk of the above-mentioned adverse outcomes remains hard to estimate (Baker and Freeman 2018; Koren et al. 2020; Li et al. 2020; Poulton et al. 2018).

Notably, breastfeeding is not considered as contraindication for treatment with MPH, since just a very small fraction of the drug is secreted in milk (making MPH undetectable in the infant’s serum) (Ornoy 2018; U.S. National Library of Medicine 2020).

## Discussion

In this paper, we provided an overview of the current knowledge about MPH in the treatment of adult patients with ADHD. We covered a broad range of issues, from the basic pharmacology to specific clinical considerations. Admittedly, a number of important items (e.g., the rationale of using MPH in ADHD co-occurring with substance use disorders [Crunelle et al. 2018; Skoglund et al. 2017], and the potential role of MPH as a cognitive enhancer [Linssen et al. 2014; Westbrook et al. 2020]) remained beyond the scope of this article.

Methylphenidate acts primarily as the DA reuptake inhibitor, at the same time securing the balance between the availability of intra-synaptic DA and the intracellular pool of the neurotransmitter (through the interactions with VMAT-2). Therefore, it is devoid of the neurotoxic properties — unlike the DA-releasing psychostimulants (e.g., methamphetamine) (Fleckenstein et al. 2009; German et al. 2015). The relatively slow entry of MPH into the brain, coupled with the likewise slow pace of the clearance, makes the drug relatively safe in terms of the risk of abuse (Volkow et al. 1995; Volkow and Swanson 2003). On a more general note, one might hypothesize that MPH and other stimulants bear the status of proverbial ‘drugs for ADHD’ — and here is why. Given the fact that ADHD represents a failure to deactivate DMN during low-salient tasks (i.e., slow, boring and unrewarding chores; in other words — the heavy duties of everyday life), MPH was notably found to be able to ‘switch the DMN off’ in the above-mentioned situations, thus increasing salience of stimuli that would otherwise end up as fuel for MW (Bozhilova et al. 2018; Liddle et al. 2011).

While reading the closing section of a paper on clinical pharmacology, one might expect a succinct discussion of ‘pros and cons of the medication in question’. However, when pondering about the rationale of using MPH for ADHD in adults, the conclusions must be drawn against the backdrop of numerous paradoxes (clinical and cultural alike) and seemingly loose ends in the research data. Here, we have the drug known for nearly 80 years and still quite ‘vague’ (Pliszka 2019; Wenthur 2016); notable for its effectiveness and yet ‘controversial’ (Cortese et al. 2018); relatively safe and mired by ‘stigma by association’ with illicit and addictive psychostimulants (e.g., methamphetamine and cocaine) (Kooij et al. 2010; Volkow and Swanson 2003). On the other hand, the persistent view of ADHD as a ‘childhood disorder of dubious validity’ further contributes to the problem of stigma (Faraone 2020; Masuch et al. 2019; Mueller et al. 2012), making the road to adequate diagnosis and treatment even more bumpy for adult individuals. And while ‘loose ends’ are hardly unique in psychiatric research in general (Alda and Hajek 2012), there are, in our opinion, some specific aspects making it particularly challenging to get one’s head around the treatment objectives in ADHD. Precisely, ADHD is an umbrella term for a set of trait-like features and a developmental trajectory alike (Asherson et al. 2016; Franke et al. 2018; Fredriksen et al. 2013). For this reason, the day-to-day adverse consequences of ADHD are not limited to various social, academic, and occupational circumstances of the individual’s life (Roselló et al. 2020). In fact, as neatly coined by Faraone et al. (2015), ‘the disorder directly affects perceptions of well-being’ — the statement which, from the standpoint of everyday clinical practice, translates into a mind-boggling spectrum of patient-important outcomes (Bölte et al. 2018). Therefore, it is not possible to evaluate the utility of MPH (or any other medication for ADHD) on the grounds of impact on the core symptoms only; nor is it plausible to pre-define any ‘strict indications’ for initiating the pharmacotherapy. In general terms, treatment choices for ADHD in adults are driven by the accompanying functional impairment and the individual preferences of the patient. Of particular note, as emphasized in the recent ‘Canadian ADHD Practice Guidelines’ (2018): ‘the high morbidity of ADHD makes it important that we also weigh the risk of not treating ADHD’. This idea was further elucidated by Fredriksen et al. (2013): ‘Ultimately, the treatment goal for ADHD, whether initiated during childhood or adulthood should be, not only temporary symptom relief, but also the establishment of a more favorable long-term developmental trajectory’.

Methylphenidate along with other stimulants (i.e., amphetamine derivatives) is recommended as the medication of choice for ADHD in adults (National Institute for Health and Care Excellence 2018; Wong et al. 2019). Even though MPH seems to lag behind amphetamines in terms of efficacy against the core ADHD symptoms (Cortese et al. 2018), the SMD value of 0.5 denotes moderate (and certainly decent — as compared to numerous other psychiatric and non-psychiatric drugs [Leucht et al. 2012]) improvement. In addition, it is hard to overemphasize the importance of the recent finding by Chang et al. (2020), suggesting that treatment with psychostimulants (including MPH) is linked to lower rates of suicide attempts in patients with ADHD across the age spectrum.

The most common MPH-related adverse events are loss of appetite and insomnia (with the risk of the latter moderated by the formulation used and the treatment regimen Ginsberg et al. 2014a; Stein et al. 2012; Wynchank et al. 2017). Of note, the side effects are usually relatively mild and transitory (or at least clinically manageable). Therefore, the methodological controversies around the rationale behind treatment with MPH seem to be driven primarily by the lack of consensus about the optimal ways of evaluating the relative weight of the outcomes (Cortese 2018; Guyatt et al. 2011). Nevertheless, given the recent developments around the research on the core outcome sets for ADHD, this limitation may soon become obsolete (Bölte et al. 2018).

Still, the available evidence base on the long-term outcomes of treatment with MPH is too patchy to draw firm conclusions regarding the overall risk-to-benefit balance (Cortese 2020; Faltinsen et al. 2019; Franke et al. 2018; Volkow and Swanson 2013). Interestingly enough, it was recently discovered that the therapeutic effects of MPH in adults with ADHD might be relatively stable over time (in spite of the prior treatment discontinuation). The mechanisms behind this phenomenon are unknown (Lam et al. 2019). While the finding remains in line with the hypothesis of ‘MPH-related improvement in neurodevelopmental trajectory’ (Fredriksen et al. 2013), it is hard to reconcile with the ‘on–off’ pharmacokinetics of MPH (Patrick et al. 2005).

The current clinical guidelines provide limited support regarding the specific strategies of MPH titration in adult patients (Bolea-Alamañac et al. 2014a, b; Cortese et al. 2018; Huss et al. 2017; National Institute for Health and Care Excellence 2018). It is hardly surprising, given the paradox that the optimal dosage regimen for ADHD (in children and adults alike) has not been determined yet (Ching et al. 2019; Cortese et al. 2018; Lam et al. 2019). Notably, the network meta-analysis by Cortese et al. (2018) provided with data suggestive for the linear dose–response relationship in adults receiving MPH, yet it is unclear how this finding would specifically translate into clinical practice (Arnett and Stein 2018). In the position paper by members of the International Multicentre Persistent ADHD Collaboration, it was implied that ‘doses around 1 mg/kg [per body mass] of methylphenidate are correlated with better efficacy, yet are rarely achieved in studies of adult patients’ (Franke et al. 2018).

Let us conclude with an epidemiological note about ADHD in adults as an important ‘blind spot’ in psychiatry. Being an important source of chronic stress, ADHD is a significant risk factor for anxiety and depressive disorders (Ahnemark et al. 2018), and while psychiatric comorbidities are a rule rather than an exception among patients with ADHD (Franke et al. 2018; Katzman et al. 2017; Kooij et al. 2019a), they do not necessarily represent ‘coexisting-but-separate’ clinical issues. Actually, accompanying mental disorders oftentimes reflect downstream effects of ADHD. There is substantial body of evidence to suggest that unrecognized or misdiagnosed ADHD is an important contributor to the problem of suboptimal treatment outcomes in the broad population of patients with mental health issues (Ginsberg et al. 2014b). Hence, there is no doubt that adults with ADHD should be adequately treated, while MPH appears to be a relatively effective and safe therapeutic option.

## Data Availability

Not applicable (given the fact that no new data were created or analyzed for the purpose of this review paper).
